# Cristae: bridging bioenergetic hubs and compartmental barriers in mitochondrial homeostasis

**DOI:** 10.1038/s41419-026-08779-x

**Published:** 2026-04-25

**Authors:** Jin Rao, Qian-qian Wan, Lei Chen, Rong-bing Tang, Daniya Killedar, Franklin Tay, Li-na Niu

**Affiliations:** 1 State Key Laboratory of Oral & Maxillofacial Reconstruction and Regeneration, National Clinical Research Center for Oral Diseases, Shaanxi Key Laboratory of Stomatology, Department of Prosthodontics, School of Stomatology, The Fourth Military Medical University, Xi’an, China; 2 Department of Stomatology, Air Force Medical Center, Beijing, China; 3 School of Stomatology, Lanzhou University, Lanzhou, China; 4 The Dental College of Georgia, Augusta University, Augusta, GA USA

**Keywords:** Cell biology, Mechanisms of disease, Mitochondria

## Abstract

Mitochondrial cristae are intricately folded structures of the inner mitochondrial membrane that play essential roles in cellular energy production, metabolic regulation, and compartmentalization. Far from being passive folds, cristae are dynamic, functional entities central to mitochondrial bioenergetics. Their architecture maximizes membrane surface area and spatially organizes protein complexes to enhance oxidative phosphorylation and adenosine triphosphate (ATP) synthesis. The compartmentalized structure of cristae also establishes functional barriers that help maintain localized proton gradients, optimize metabolic reactions, and contribute to mitochondrial stability. These dual roles in energy transformation and spatial segregation underscore the importance of the cristae in supporting cellular homeostasis. The structural design and lipid composition of cristae with enrichment in cardiolipin also reflect their bacterial ancestry, revealing an evolutionary continuity from prokaryotic bioenergetic systems to eukaryotic organelles. Moreover, dynamic remodeling of cristae in response to stress, nutrient availability, and developmental cues highlights their adaptability in regulating mitochondrial performance and signaling pathways. Disruption of cristae architecture is increasingly implicated in neurodegenerative, cardiovascular, and metabolic diseases due to impaired ATP synthesis and compromised mitochondrial integrity. This review examines emerging insights into the organization, composition, and regulatory mechanisms of the cristae, emphasizing their role as both bioenergetic engines and protective compartments. Understanding the complex interplay between cristae structure and mitochondrial function may illuminate novel strategies for restoring mitochondrial health and targeting diseases linked to mitochondrial dysfunction. Cristae represent an evolutionary innovation that bridges structure and function, enabling the mitochondria to meet the multifaceted demands of the eukaryotic cell.

## Facts

- Cristae represent an evolutionary structural adaptation that utilizes endosymbiotically acquired bacterial characteristics to facilitate mitochondrial autonomy within eukaryotic systems.
- Cristae serve dual functions as bioenergetic hubs and compartmental barriers that enable rapid mitochondrial functional reprogramming.
- The non-bilayer molecular geometry of cardiolipin is the mechanistic basis of cristae integrity and protein networks, including the MICOS complex, OPA1, and ATP synthase, hierarchically organize cristae to coordinate their architecture and function.

## Open questions

- What selective forces drove the emergence of cristae folds in early endosymbionts—was it primarily for bioenergetic optimization, evasion of host immune surveillance, or compartmentalization of toxic byproducts?
- Does the unique conical geometry of cardiolipin promote liquid-liquid phase separation (LLPS) by lowering the critical transition concentration of cristae-resident proteins, and is this process modulated by membrane oxidation states?
- Can quantitative cristae morphometrics serve as preclinical biomarkers for diseases linked to mitochondrial dysfunction?

## Introduction

Mitochondria are often described as cellular powerhouses. These intracellular organelles supply eukaryotes with energy in the form of adenosine triphosphate (ATP) to serve their cellular needs [1]. Recent decades have seen an evolution in our understanding of the important role of the mitochondria in signaling, biosynthesis, and cell fate regulation apart from mere energy provision [2–4]. This complexity reflects the idea behind the endosymbiotic theory that mitochondria began as free-living bacteria that formed a mutually beneficial partnership with early eukaryotic cells. Through evolution, mitochondria became deeply embedded within their host cells, gradually changing in both form and function to support energy production and essential cellular processes [5–8].

A unique structural feature that makes mitochondria so effective is the inner mitochondrial membrane (IMM). The IMM folds inward to form elaborate structures called cristae [9]. Unlike the outer mitochondrial membrane (OMM), which possesses a lipid composition similar to that of eukaryotic plasma membranes, the IMM is uniquely enriched in cardiolipin and lacks cholesterol, a phospholipid profile that strongly resembles that of bacterial membranes and reflects its endosymbiotic evolutionary roots [10–12]. These cristae are essential for ATP production and for maintaining mitochondrial homeostasis. However, cristae are not static structures, and they undergo active remodeling that determines cell fate. The widening of crista junctions (CJs) is a decisive event that mobilizes cytochrome c from the cristae lumen to initiate apoptosis. Following this remodeling, severe loss of inner membrane integrity can further lead to the release of mitochondrial DNA (mtDNA) and reactive oxygen species (ROS), amplifying inflammatory signaling and necrotic cell death [13–15].

**Fig. 1:**
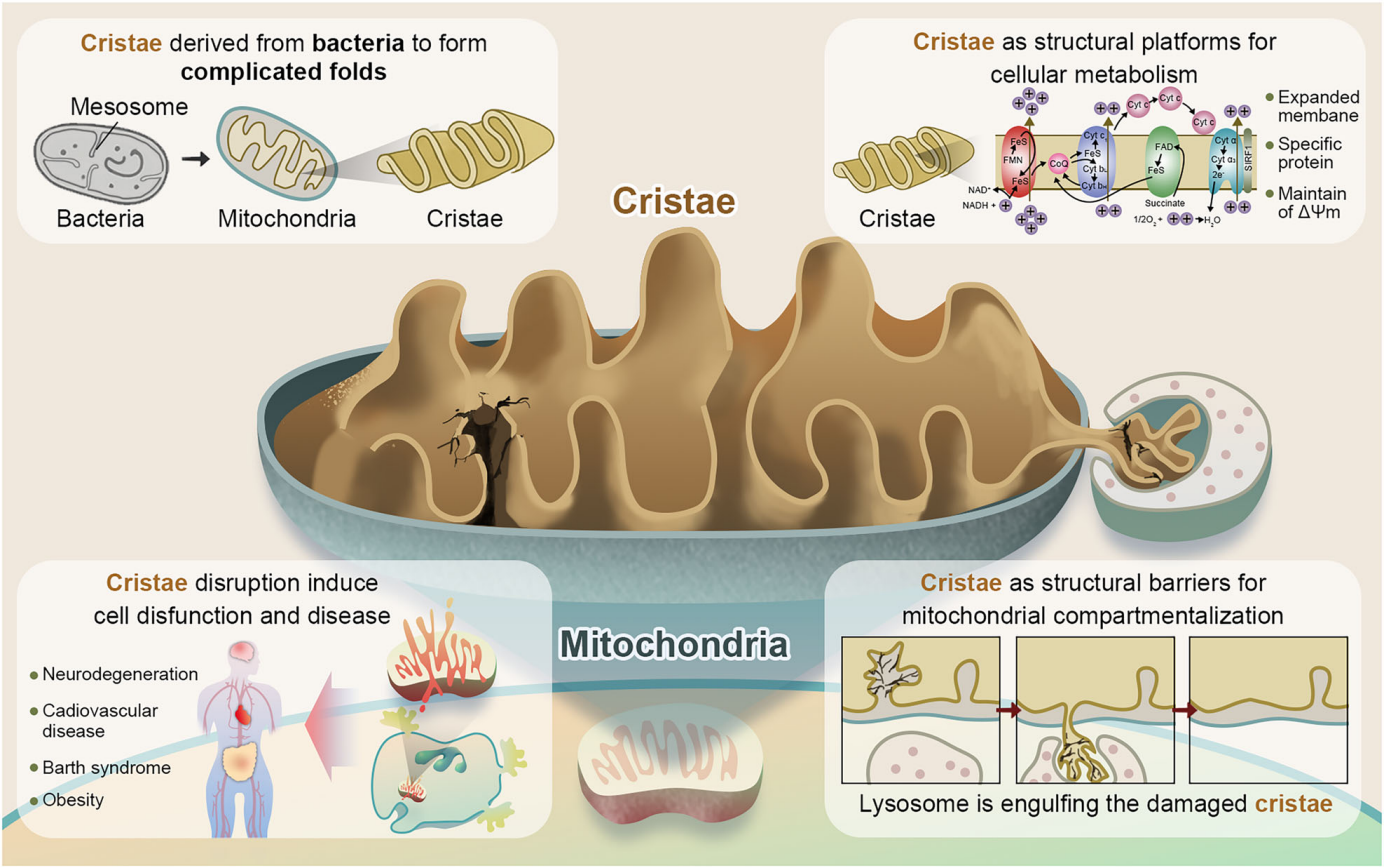
The dual functionality of mitochondrial cristae - active bioenergetic sites and structural barriers for compartmentalization. Cristae create a highly organized and compartmentalized environment that houses protein complexes and enzymes to serve two primary functions: bioenergetic hubs and compartmental barriers, which reflects adaptations inherited from their bacterial ancestors during the shift to life inside eukaryotic cells.

Although mitochondria are fully integrated into eukaryotic cells, their structure and behavior still resemble that of ancient bacteria enclosed by the OMM. Their bacterial roots have not been erased by evolution. Instead, many of those original features within the cristae remain essential for function. Cristae act as both sites for energy production and barriers that create separate spaces within the mitochondrion. These roles reflect adaptations inherited from their bacterial ancestors during the shift to life inside eukaryotic cells. Understanding the structure and behavior of cristae helps clarify how mitochondria support metabolism, coordinate signaling, and respond to stress. Such information offers new insights on mitochondrial diseases and their impact on overall cellular health.Fig 1.

## Cristae: shaping mitochondrial function through structural organization

The idea that structure supports function is a recurring theme in biology. Mitochondrial cristae offer a clear example of this principle in action. These elaborate folds within the IMM are carefully sculptured for energy production and cellular organization [16]. Mitochondria are enclosed by two distinct membranes, each with its own specialized structure and function. The OMM acts as a boundary separating the organelle from the cytosol. The IMM has two regions: one that runs alongside the outer membrane, known as the inner boundary membrane, and another that folds inward to form the cristae. The cristae extend into the mitochondrial matrix to create a highly-organized internal landscape that supports the many demands placed on this essential organelle [17].

### Tools for revealing cristae architecture

Since cristae were first identified under the electron microscope, they have been perceived as more than just simple membrane folds. Early studies viewed them as static structural features [18]. The advent of imaging techniques, such as cryo-electron microscopy and fluorescence lifetime imaging microscopy has revealed a much more dynamic picture [19–23]. Cristae are now recognized as flexible, highly-organized structures that continuously adapt to the energy needs and metabolic state of a cell. Molecular dynamics simulations [24] and mathematical modeling [25, 26] have improved this understanding by showing how cristae can shift shape and function in response to different physiological conditions [27, 28].

More recently, live-cell imaging has allowed scientists to directly observe these changes in real time to capture remodeling of the IMM during mitochondrial fusion and fission [29–31]. These studies show that cristae are anything but static. Their ability to reshape in response to shifting energy demands is vital for maintaining efficient ATP production and metabolic flexibility [18, 32]. Deep learning tools have begun to model and predict cristae behavior with remarkable precision. These artificial intelligence technologies have opened new vistas for understanding how these structures respond to stress or dysfunction [33, 34].

**Fig. 2:**
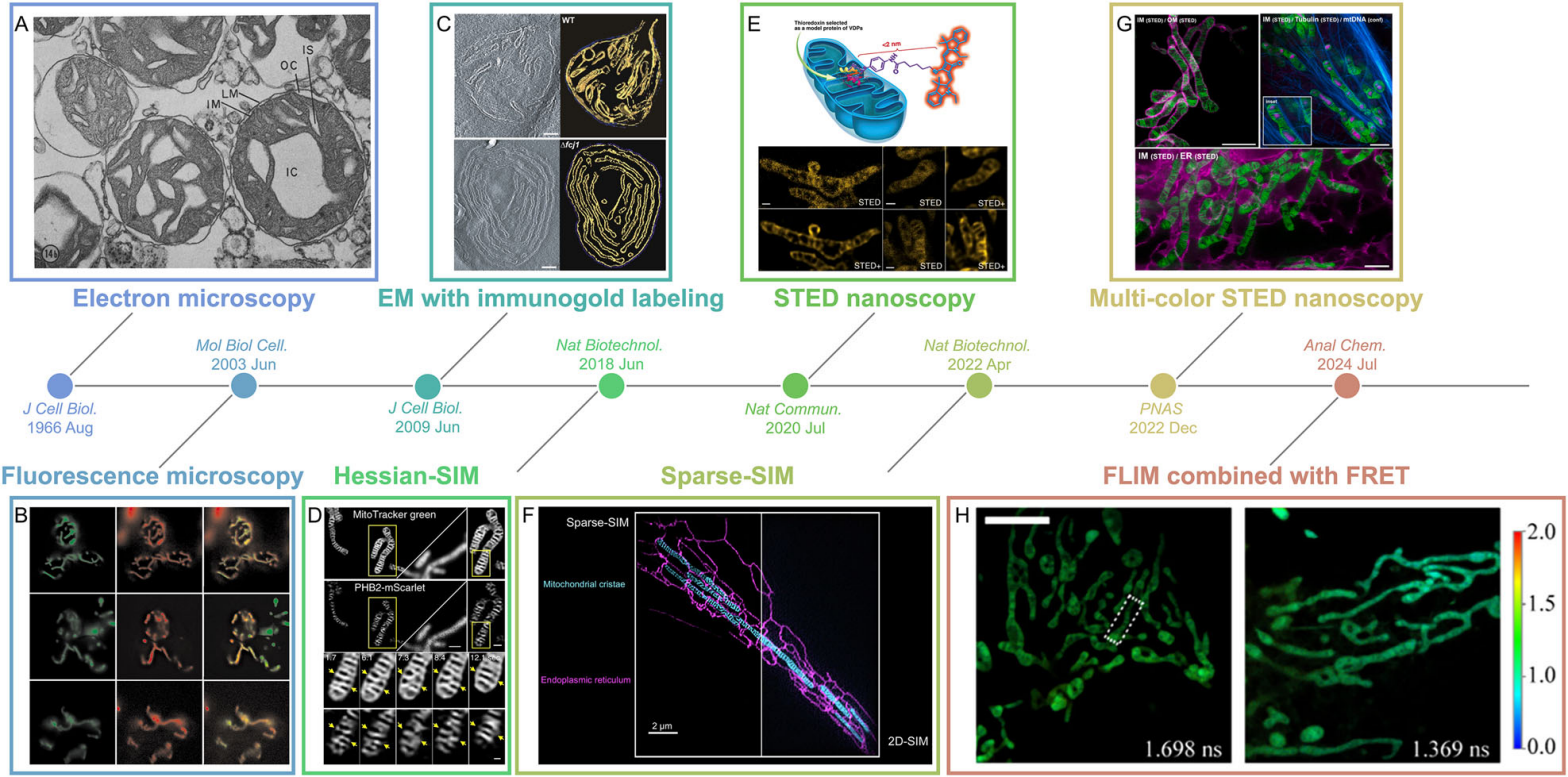
Timeline of advancing methodologies for visualizing cristae architecture and dynamics. **A** First clear visualization of cristae structure using electron microscopy, adapted from Hackenbrock CR, J Cell Biol, with permission via Copyright Clearance Center. **B** Early observation of mitochondrial fusion events enabled by fluorescence microscopy, adapted from Sesaki H et al., Mol Biol Cell, with permission via Copyright Clearance Center. **C** Identification of the MICOS complex subunit essential for cristae organization using immunogold labeling, adapted from Rabl R et al., J Cell Biol, with permission via Copyright Clearance Center. **D** Rapid, artifact-free imaging of cristae dynamics achieved by Hessian-SIM, adapted from Huang X et al., Nat Biotechnol, with permission via Copyright Clearance Center. **E** Real-time imaging of cristae morphology in live cells using a modified squaraine probe with STED nanoscopy, adapted from Yang X et al., Nat Commun, with permission via Copyright Clearance Center. **F** High-speed capture of detailed cristae structures in living mitochondria via Sparse-SIM, adapted from Zhao W et al., Nat Biotechnol, with permission via Copyright Clearance Center. **G** 3D reconstruction of cristae networks within intact mitochondria using multi-color live-cell STED, adapted from Liu T et al., Proc Natl Acad Sci U S A, with permission via Copyright Clearance Center. **H** Simultaneous super-resolution monitoring of cristae architecture and mitochondrial membrane potential using FRET combined with FLIM, adapted from Peng F et al., Anal Chem, with permission via Copyright Clearance Center.

Cristae fold into highly-convoluted structures that adapt dynamically to cell type, physiological state, and metabolic demand [35]. For instance, in high-energy demand tissues like cardiomyocytes, cristae are densely packed and arranged in lamellar stacks to maximize the surface area for oxidative phosphorylation (OXPHOS). In contrast, mitochondria in steroidogenic cells often exhibit tubular or vesicular cristae, a specialized architecture that facilitates the import of cholesterol for steroid hormone synthesis [36]. Their lamellar, tubular, parallel, or network-like configurations create a complex internal landscape essential for mitochondrial function [37]. This structural diversity is more than a morphological feature; it plays a central role in optimizing energy production and enabling metabolic flexibility [38]. As imaging and analytical tools continue to advance, the ability to visualize, measure, and even manipulate cristae structures unlocks new possibilities for examining how cells manage energy at a detailed level (Fig. 2). These insights may also guide the development of new approaches to treating mitochondrial dysfunction.

### Conical phospholipids and their role in cristae structure

An essential feature of cristae is their ability to support energy production by creating a compact, specialized environment with distinct mechanical properties; this function is largely driven by their unique lipid scaffolds [39]. Similar to other membranes, mitochondrial IMM has a tightly-regulated lipid composition. However, it also contains unique features that set it apart. Phosphatidylcholine and phosphatidylethanolamine make up about 75% of IMM lipids. However, cristae are especially enriched in conical phospholipids, such as phosphatidylethanolamine and cardiolipin [12]. These lipids act as structural scaffolds that are responsible for stabilizing the curved folds of the membrane [40].

Cardiolipin is the hallmark lipid of cristae that is essential for maintaining their structure and function. This lipid component is almost exclusive to the inner membranes of non-photosynthetic eukaryotes [41]. Cardiolipin comprises roughly 15–20% of the phospholipid mass in this region [42, 43]. Its conical shape is created by four acyl chains that account for the constricted curvature of the cristae [44–46]. This curvature increases membrane surface area, which is essential for housing large numbers of respiratory proteins and promoting efficient ATP synthesis [47, 48]. Cardiolipin also plays a functional role by anchoring respiratory complexes and supercomplexes in place [49]. This spatial organization improves electron transport efficacy by minimizing the distance between protein complexes. Altogether, cardiolipin creates a responsive lipid-protein framework that enhances the speed and precision of energy conversion in the cristae, especially during high metabolic demands.

The role of cardiolipin in shaping cristae structure and function reflects its deep evolutionary roots, tracing back to bacterial membranes [50]. In these prokaryotic ancestors, cardiolipin helped stabilize membranes under stress and supported dense protein environments. This essential function is retained in mitochondrial cristae today [51]. To accommodate this dense protein environment, cardiolipin undergoes a critical maturation process known as “remodeling.” This enzymatic cycle, primarily driven by the transacylase Tafazzin, replaces the diverse acyl chains of nascent cardiolipin with specific unsaturated fatty acids (typically linoleic acid) [52, 53]. This remodeling transforms cardiolipin into a conical-shaped molecule that significantly enhances membrane fluidity and curvature elasticity. By minimizing the biophysical tension caused by high curvature, remodeled cardiolipin allows the cristae membrane to withstand the lateral pressure exerted by protein crowding, thereby maintaining structural integrity during metabolic fluctuations [45]. For instance, classic ultrastructural studies have shown that cristae transition from a relaxed “orthodox” configuration in resting conditions to a “condensed” state with expanded intracristal spaces during active ATP synthesis, thereby increasing the local capacity for proton storage [18, 54]. Similarly, during the metabolic reprogramming of T cells, activation triggers a rapid tightening of cristae to ramp up ATP production[55]. This structural diversity transcends mere morphological characteristics, serving as a pivotal factor in enhancing energy production and supporting metabolic flexibility [56–59].

Although most research efforts focused on the protein complexes involved in energy production, the role of lipids like cardiolipin deserves equal attention. As a conical phospholipid, cardiolipin acts as an “architect” of the cristae, building a lipid scaffold that supports both the structure and function of mitochondria. Inheriting this design from ancient bacteria, cardiolipin gives cristae the adaptability, strength, and organization required to sustain high levels of energy output.

### Molecular networks in cristae regulation

**Fig. 3:**
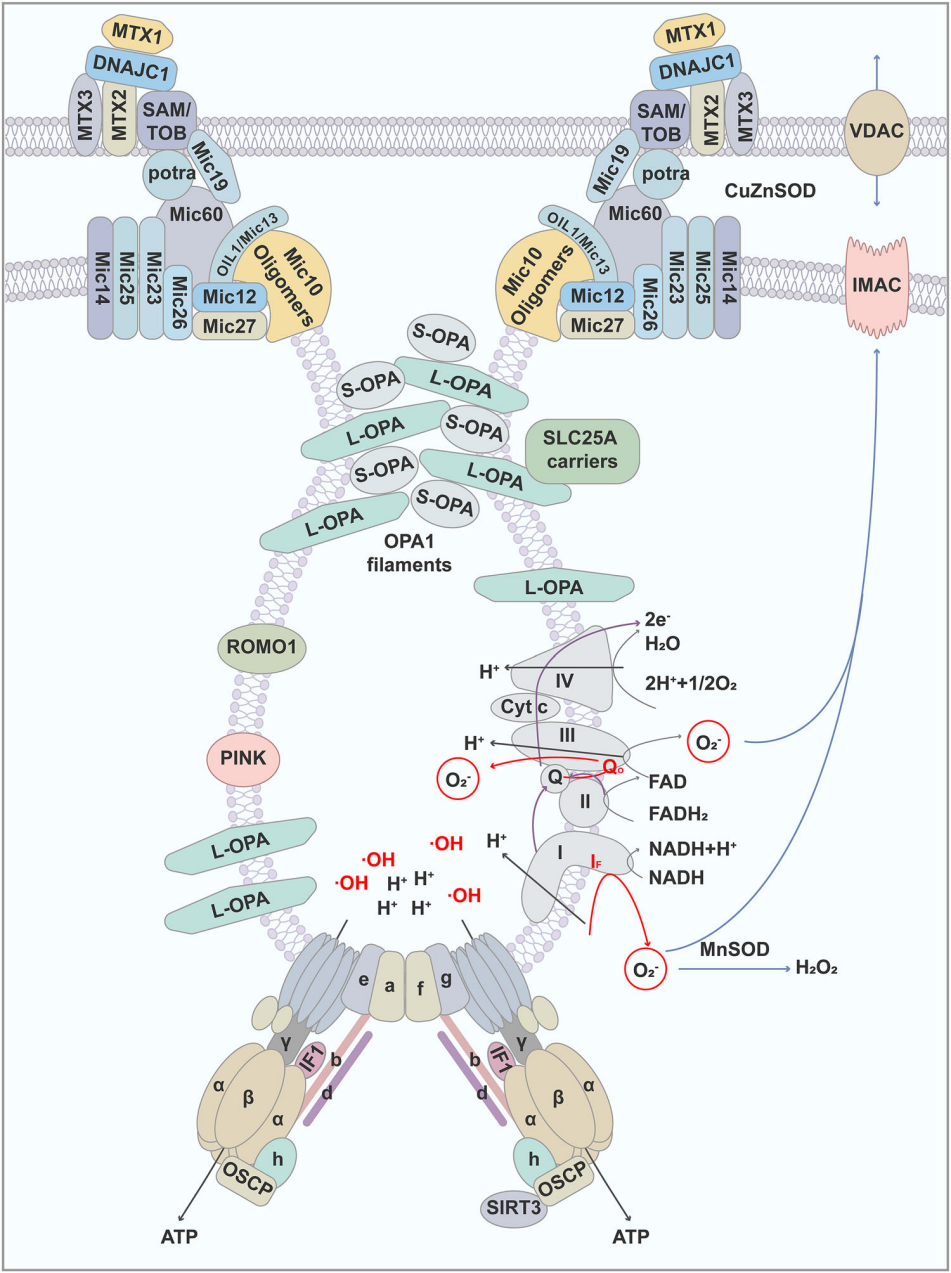
Molecular architecture and functional compartmentalization of mitochondrial cristae. This schematic illustrates the specialized spatial organization of protein complexes that maximize bioenergetic efficiency and maintain structural integrity. Top/Junctions, The MICOS complex anchors the CJs, bridging the inner boundary membrane with the cristae membrane to stabilize the compartment. Middle/Lumen, OPA1 filaments (comprising long and short isoforms) act as structural staples to regulate CJ tightness, while SLC25A carriers facilitate selective metabolite transport across the membrane. Bottom/Tips, F₁F₀-ATP synthase dimers assemble at the highly curved cristae ridges, acting as proton sinks to optimize ATP generation. Right/Bioenergetics, The ETC complexes (I–IV) are embedded within the cristae membrane, establishing the proton gradient (H^+^) that drives oxidative phosphorylation. Additionally, cristae serve as hubs for ROS management, where reactive species (superoxide O_2_^-^ and hydroxyl radicals ·OH) are strictly regulated by localized antioxidant enzymes and metabolic sensors like SIRT3 to ensure homeostasis.

The stability and dynamic organization of cristae are essential for maintaining both the structural integrity and functional efficiency of mitochondria. Proper cristae architecture is vital for effective distribution of energy within the cell, directly impacting metabolic performance and overall cellular health. This architecture is governed by a sophisticated interplay of protein complexes and metabolic cofactors (Fig. 3).

#### MICOS: the structural anchor

A central player in controlling cristae shape and organization is the mitochondrial contact site and cristae organizing system (MICOS) complex [60]. Strategically positioned at CJs, this complex harbors narrow openings that connect the cristae to the inner boundary membrane of the IMM [61, 62]. By maintaining these junctions, the MICOS complex creates distinct compartments that separate mtDNA from the surrounding matrix. This spatial organization helps stabilize mtDNA and supports its proper replication, both of which are vital for sustained mitochondrial function [63]. Furthermore, MICOS ensures accurate protein import into cristae for executing energy-related pathways, such as OXPHOS [64].

#### OPA1: the dynamic remodeler

However, MICOS does not work alone; it functions in close coordination with optic atrophy 1 (OPA1), a dynamin-related GTPase that acts as the master regulator of inner membrane fusion and cristae architecture. Unlike other dynamins that primarily pinch membranes, OPA1 forms oligomers that act as a structural staple to keep CJs mechanically tight. This function prevents the leakage of cytochrome c and maintains the chemiosmotic gradient. Crucially, the activity of OPA1 relies heavily on its interaction with membrane lipids. A recent breakthrough revealed that the paddle domain of OPA1 extensively engages with the acyl chains of cardiolipin. This lipid-protein interaction anchors OPA1 to the membrane and induces the conformational changes required for membrane remodeling and fusion [65]. Thus, cardiolipin is not just a passive dock but an active cofactor that empowers OPA1 to reshape cristae in response to metabolic cues.

#### The interconnected protein-lipid network

These key players operate within a broader network of interacting proteins. The MICOS complex collaborates with OPA1 and F₁F₀-ATP synthase (located at cristae tips) to form specialized protein-lipid domains with cardiolipin. These domains are critical for shaping, stabilizing, and maintaining cristae membranes in response to the cell’s energy demands [66]. This coordination is especially important during cellular stress, when the preservation of cristae structure and mitochondrial membrane potential (ΔΨm) becomes essential [67]. Additionally, the MICOS complex works with the translocase of the inner membrane 22 (TIM22) complex to ensure accurate import of carrier proteins [68], an essential process for maintaining mitochondrial activity and energy metabolism. Together, these proteins form an interconnected network that upholds cristae structure and enables the mitochondria to adjust flexibly to shifting energy needs of the cell [69].

#### Metabolic regulation by NAD^+^

The maintenance of cristae function depends equally on the metabolic cofactors that regulate their activity. While protein complexes establish the physical architecture, nicotinamide adenine dinucleotide (NAD^+^) plays a central role in stabilizing this architecture through redox regulation. As the primary electron acceptor in oxidative metabolism, NAD^+^ governs the activity of Complex I, thereby influencing respiratory flux and ATP output [70]. Moreover, NAD^+^ availability modulates the activity of sirtuins (e.g., SIRT3) and OPA1, directly linking metabolic status to cristae remodeling and fusion machinery [71–73]. During aging or metabolic stress, NAD^+^ depletion leads to altered cristae morphology, impaired membrane potential, and mitochondrial fragmentation [74–76]. Restoration of NAD^+^ levels through supplementation with precursors, such as nicotinamide mononucleotide (NMN) or nicotinamide riboside (NR) has been shown to preserve cristae architecture, enhance oxidative phosphorylation, and protect mitochondrial integrity in multiple tissue types [77].

By exploring the complex interactions of these cristae-associated elements (Table 1), researchers are uncovering the mechanisms that govern organelle organization [78]. Many of these molecules are thought to be absent in the ancestral bacteria from which mitochondria evolved. Instead, they likely emerged as evolutionary adaptations to meet the higher energy demands of eukaryotic cells. These evolutionary adaptations drove the development of specialized protein machineries, such as the MICOS complex, which originated from bacterial membrane-organizing proteins to maintain the intricate architecture of cristae [79]. Understanding this structural evolution provides key insights into how eukaryotic cells optimized mitochondrial bioenergetics to sustain complex life functions.

**Table 1:** Key molecular determinants of cristae architecture and regulation.

Key regulator	Type & localization	Molecular mechanism	Biological function & Impact	Key interactions	Refs
Cardiolipin	Lipid scaffold the IMM (especially enriched in cristae)	• Conical shape: 4 acyl chains create curvature to accommodate dense protein packing. • Remodeling: tafazzin replaces acyl chains with linoleic acid to enhance fluidity and minimize membrane tension.	• Structural architect: stabilizes curved folds and maintains structural integrity. • Bioenergetic hub: anchors respiratory supercomplexes to improve electron transport efficiency. • Adaptability: allows transition between “orthodox” and “condensed” states.	Interacts with OPA1 (paddle domain) and respiratory supercomplexes.	12, 39, 40, 44-49, 52-54, 65
MICOS Complex	Structural anchor CJs	• Forms narrow openings connecting cristae to the inner boundary membrane. • Evolutionary origin from bacterial membrane-organizing proteins.	• Compartmentalization: separates mtDNA from the matrix to support replication. • Protein import: facilitates accurate import for OXPHOS pathways. • Stability: acts as a static anchor for cristae shape.	Collaborates with TIM22, OPA1, and ATP synthase.	60–64, 68, 79
OPA1	Dynamic remodeler CJs & the IMM	• Oligomerization: Forms a structural staple to mechanically tighten CJs. • Lipid binding: paddle domain engages with cardiolipin acyl chains to induce membrane remodeling.	• Fusion & Integrity: master regulator of inner membrane fusion. • Barrier function: prevents cytochrome *c* leakage and maintains chemiosmotic gradient. • Responsiveness: reshapes cristae based on metabolic cues.	Activity depends on Cardiolipin interaction; regulated by NAD^+^/Sirtuins.	65
F₁F₀-ATP Synthase	Shaping complex Cristae tips	• Dimerization/Oligomerization (implied in context of curvature).	• Tip formation: stabilizes the highly curved tips of cristae. • Energy production: directly executes ATP synthesis.	Works within the protein-lipid domains formed by MICOS and Cardiolipin.	66, 67
NAD^+^	Metabolic cofactor Matrix / Microenvironment	• Redox regulation: acts as primary electron acceptor. • Signaling: modulates activity of Sirtuins (e.g., SIRT3) and OPA1.	• Metabolic-structural link: couples metabolic status to cristae morphology. • Protection: prevents mitochondrial fragmentation and preserves ΔΨm under stress.	Regulates Complex I activity and OPA1 fusion machinery.	70–77

### Cristae dysfunction in pathological conditions

**Fig. 4:**
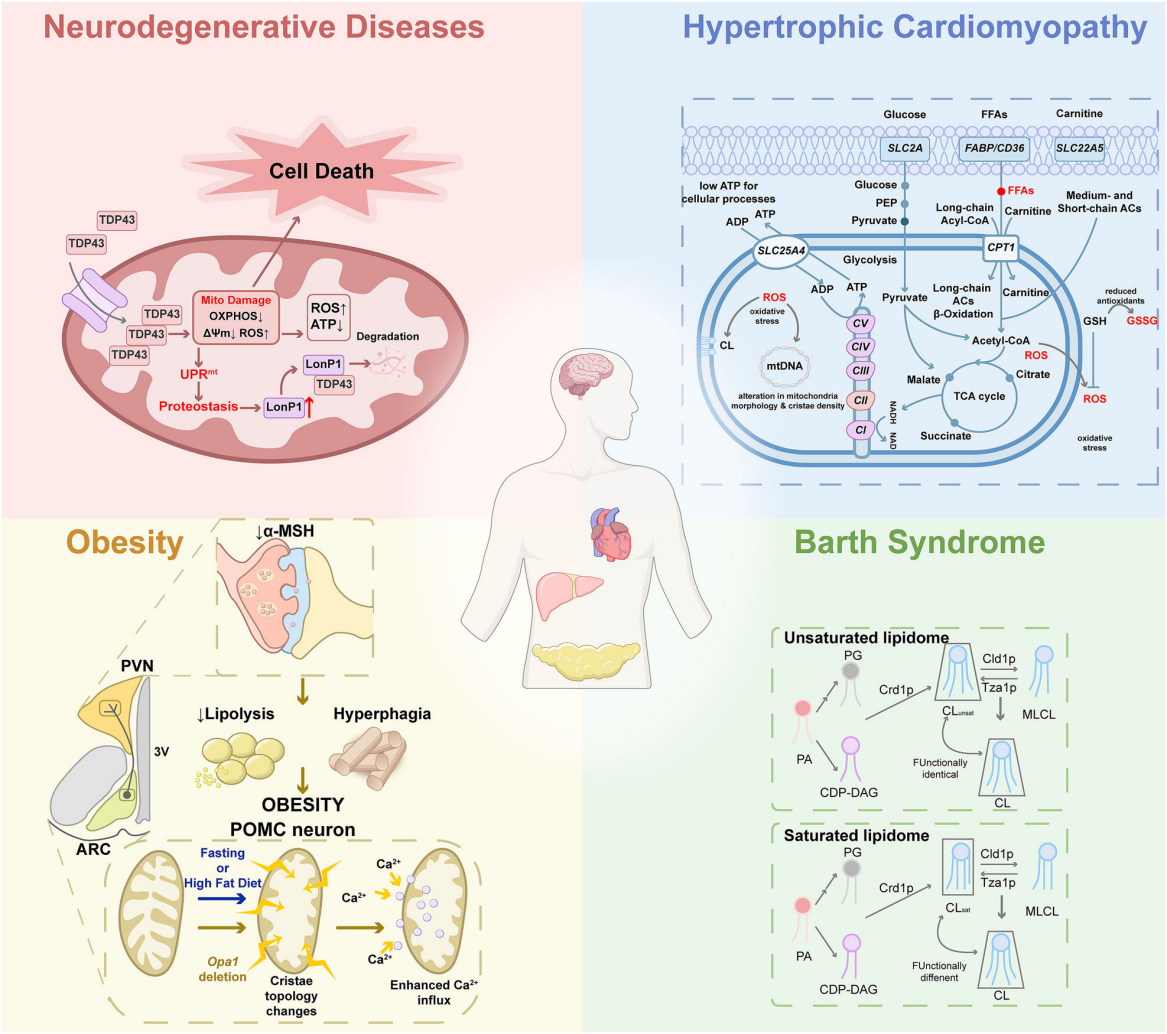
Mechanisms linking cristae disruption to systemic pathologies in high-energy tissues. Structural instability of cristae drives energy depletion and cell death, serving as a central pathogenic mechanism in diverse diseases. In neurodegenerative diseases, mitochondrial accumulation of TDP-43 induces cristae damage and triggers the mitochondrial unfolded protein response (ref. 81). In hypertrophic cardiomyopathy, metabolic stress and elevated ROS production compromise cristae integrity—damaging mtDNA and cardiolipins—which leads to myocardial energy deprivation (ref. 84). In obesity, nutritional states regulate OPA1 expression in POMC neurons; OPA1 deficiency disrupts cristae topology and mitochondrial Ca^2+^ handling, thereby impairing systemic metabolic regulation (ref. 67). In Barth syndrome, defects in the remodeling of cardiolipin (the signature lipid of the inner mitochondrial membrane) result in abnormal cristae architecture and mitochondrial dysfunction (ref. 90).

The structural integrity of cristae is inextricably linked to mitochondrial performance. When this architecture is disrupted, the consequences extend beyond bioenergetic inefficiency to compromise cell survival. Before discussing specific pathologies, it is essential to understand how cristae architecture dictates cell survival. The cristae remodeling model establishes that the release of apoptogenic factors is structurally controlled. Most cytochrome c is sequestered within the cristae folds and cannot freely diffuse into the cytosol due to the narrow bottlenecks formed by CJs. During early apoptosis, the oligomers of OPA1 that maintain these junctions are disrupted, leading to CJ widening. This structural opening allows the rapid efflux of cytochrome c into the intermembrane space and subsequently into the cytosol, triggering the caspase cascade. Thus, the maintenance of cristae topology acts as a structural checkpoint that prevents accidental cell death, while its remodeling serves as a primary mechanism driving apoptosis [80]. Emerging evidence implicates cristae remodeling defects in a spectrum of pathologies. Below, we examine the mechanistic links between cristae dysfunction and specific disease categories (Fig. 4).

#### Neurodegenerative diseases

In neurons, which have high energy demands, cristae architecture is critical. Altered cristae structure impairs OXPHOS, leading to energy deficits and neuronal death. In amyotrophic lateral sclerosis (ALS), the pathological accumulation of TAR DNA-binding protein 43 (TDP-43) inside mitochondria has been shown to directly damage cristae, activating the mitochondrial unfolded protein response and compromising function [81]. Likewise, mutations in cristae-shaping proteins within the mitochondrial contact site and the MICOS complex have been linked to abnormal cristae architecture and defective electron transport chain activity in several neurodegenerative conditions [66]. Strategies that stabilize cristae morphology or enhance mitochondrial quality control are being explored. For instance, restoring NAD^+^ levels, which decline with aging, can activate sirtuins and OPA1 to preserve cristae structure and prevent neurodegeneration [82, 83].

#### Cardiovascular diseases

Cardiac tissue depends critically on efficient ATP production. Consequently, cristae disorganization is also a major contributor to mitochondrial dysfunction in cardiovascular diseases. In conditions like hypertrophic cardiomyopathy, disrupted cristae structure results in inefficient energy production in heart muscle cells that adversely affects the progression of heart failure [84]. Similar abnormalities are observed in ischemic heart disease, where damaged cristae impair ATP synthesis and further compromise myocardial tissue. Metabolic disorders, such as metabolic syndrome feature cristae alterations that reduce mitochondrial efficacy and contribute to broader cardiac dysfunction. Pyridostigmine, an acetylcholinesterase inhibitor traditionally used for myasthenia gravis, has been shown to restore cristae integrity in cardiomyopathies. Mechanistically, it activates M3 muscarinic acetylcholine receptors, initiating AMPK signaling pathway that upregulates OPA1 expression. This upregulation promotes the re-tightening of cristae junctions and enhances ATP production, effectively reversing the cristae collapse observed in metabolic stress [85].

#### Genetic disorders

Genetic disorders, such as Barth syndrome further highlight the importance of cristae integrity in mitochondrial health. Barth syndrome is caused by mutations in the TAZ gene, which encodes the transacylase tafazzin. Loss of tafazzin function prevents the remodeling of monolyso-cardiolipin (MLCL) into mature tetralinoleoyl-cardiolipin (TLCL). The resulting accumulation of MLCL and deficiency of TLCL destabilize the negative curvature of the inner membrane [86]. Since mature cardiolipin acts as a molecular glue, its absence leads to the detachment of OPA1 from the membrane and the disintegration of respiratory supercomplexes, causing loosely packed, collapsed cristae and severe metabolic failure [87].

Overall, this condition disrupts the remodeling of cardiolipin and is closely associated with abnormal cristae structures [88–90]. When cardiolipin remodeling is impaired, cristae formation becomes defective, respiratory chain complexes are destabilized, and overall mitochondrial function declines [91]. Because of the central role of cardiolipin in maintaining mitochondrial structure and bioenergetics, it has also emerged as a promising therapeutic target for restoring mitochondrial function in age-related diseases [92].

#### Metabolic disorders

Cristae remodeling is not restricted to local organellar maintenance but also governs systemic metabolism. Recent studies indicate that mitochondrial architecture in hypothalamic neurons acts as a metabolic switch regulating body weight. Specifically, in pro-opiomelanocortin (POMC) neurons, which suppress appetite and promote energy expenditure, the quantity of OPA1 and cristae tightness fluctuate with nutritional states. A pivotal study demonstrated that genetic deletion of OPA1 in these neurons causes cristae dilation and disrupts mitochondrial Ca^2+^ buffering capacity. This loss of Ca^2+^ homeostasis impairs the processing and release of α-melanocyte-stimulating hormone (α-MSH). Consequently, this central defect in cristae architecture remotely inhibits lipolysis in adipose tissue, leading to massive weight gain and obesity [67]. This finding underscores a critical link between neuronal cristae plasticity and the systemic regulation of fat mass.

Studies on mitochondrial diseases emphasize the importance of preserving cristae structure to maintain mitochondrial function. Cristae are essential for the efficiency of the electron transport chain. Disruptions in their architecture can trigger energy shortages, promote cell death, and worsen disease progression (Table 2). Protecting cristae integrity is especially critical in energy-demanding tissues like the brain, heart, and muscles, where mitochondrial performance is closely tied to overall cellular health and survival [93].

**Table 2:** Pathological mechanisms of cristae dysfunction and therapeutic targets.

Disease category	Specific condition / trigger	Molecular & structural impact on cristae	Functional consequence	Therapeutic strategy / target	Refs
General mechanism (apoptosis)	CJ widening	Disruption of OPA1 oligomers opens the “bottlenecks” at CJs.	Release of sequestered cytochrome c into cytosol → triggers caspase cascade → apoptosis.	Stabilizing CJ structural checkpoints to prevent accidental cell death.	80
Neurodegenerative diseases	Amyotrophic lateral sclerosis	Accumulation of TDP-43 inside mitochondria directly damages cristae; activates mitochondrial unfolded protein response.	Impaired OXPHOS; energy deficits; neuronal death.	Restoring NAD^+^ levels: activates sirtuins and OPA1 to preserve cristae structure.	81–83
	General neurodegeneration	Mutations in MICOS complex components.	Abnormal cristae architecture; defective ETC activity.	Enhancing mitochondrial quality control.	66
Cardiovascular diseases	Metabolic syndrome / Ischemia / Cardiomyopathy	Downregulation of OPA1; Disorganization of lipid scaffolds.	Inefficient ATP production; progression of heart failure; myocardial tissue compromise.	Pyridostigmine: inhibits AChE → activates M3 receptors → AMPK pathway → upregulates OPA1 to re-tighten CJs.	84, 85
Genetic disorders	Barth syndrome	Tafazzin deficiency: failure to remodel MLCL into mature TLCL → Accumulation of MLCL destabilizes negative membrane curvature.	Structural collapse: detachment of OPA1; disintegration of respiratory supercomplexes; severe metabolic failure.	Targeting cardiolipin remodeling to restore the lipid scaffold and bioenergetics.	86–92
Metabolic disorders	Obesity	OPA1 deficiency in POMC neurons → Cristae dilation (loss of tightness).	Impaired mitochondrial Ca^2+^ buffering → reduced α-MSH release → inhibition of adipose lipolysis.	Targeting central OPA1 or restoring neuronal Ca^2+^ homeostasis.	67

## Cristae as structural platforms for cellular metabolism

### Hubs of respiratory activity

Cristae play a central role in cellular energy metabolism, particularly by supporting OXPHOS and ATP production. This section outlines five features that make cristae the functional core of mitochondrial respiration.

#### Vast membrane surface area

The structural configuration of cristae plays a vital role in mitochondrial energy metabolism by significantly expanding the surface area of the IMM. This function is essential for accommodating the dense array of proteins involved in energy conversion [94, 95].

Intricate folding increases the localization of enzyme components, such as ATP synthase and the respiratory chain complexes. This feature enhances both the distribution and functional interaction of these enzymes [96]. The expanded membrane surface offers more sites for OXPHOS, thereby boosting the efficiency of proton pumping and ATP synthesis [97, 98]. This spatial optimization enables a more robust ATP output that is critical for sustaining high energy demands across various cellular functions. The proximity of enzyme complexes within the folded membrane also improves the speed and precision of energy transfer to minimize losses and maximize output [99]. This strategic architecture ensures that cristae support mitochondrial energy production that is responsive to cellular needs.

### Efficient and organized protein complexes

The efficiency of cristae is significantly enhanced by the precise spatial arrangement of protein complexes within their folds. Instead of being randomly distributed, OXPHOS components including ATP synthase are strategically organized into highly ordered clusters [100]. This structural alignment reduces energy dissipation that is common in less organized systems, and facilitates more efficient proton flow across the mitochondrial membrane [101]. By positioning super-complexes near ATP synthase, cristae optimize proton transfer to improve ATP production [102].

This organization is essential for maintaining high energy output in cells with elevated metabolic demands. By minimizing energy loss and maximizing reaction efficiency, cristae structure supports the role of mitochondrion as the cell’s primary energy producer. The deliberate arrangement of protein complexes reflects the highly specialized architecture of mitochondria in sustaining cellular function and vitality.

### Maintenance of proton gradient

The ability of cristae to sustain a high rate of ATP synthesis relies on their capacity to act as localized proton traps. Unlike a uniform gradient across the entire inner membrane, the intracristal space functions as a specialized reservoir where protons are concentrated. The highly folded architecture minimizes the diffusion distance between the proton pumps (Complexes I, III, and IV) located on the cristae slopes and the ATP synthase dimers situated at the cristae tips [103]. This “source-to-sink” proximity creates a steep local pH gradient and an enhanced proton motive force specifically around the ATP synthase, forcing protons to flow through the catalytic motor rather than dissipating into the bulk matrix. This kinetic coupling ensures that ATP generation remains efficient even when the global mitochondrial membrane potential fluctuates [104]. Disruption of this gradient significantly reduces the efficacy of ATP production, ultimately affecting the energy supply of the cell [105].

Beyond its catalytic role in ATP production, the supramolecular organization of ATP synthase is fundamental to cristae architecture. The enzyme complexes assemble into V-shaped dimers that further organize into long oligomeric rows, acting as a molecular sink to induce the high positive membrane curvature required for cristae rim formation. Disruption of these oligomerization subunits (e.g., subunits e and g) leads to the loss of cristae tips and the formation of aberrant onion-like inner membrane structures. This shape-determining function is evolutionarily conserved; for instance, in Apicomplexan parasites like Toxoplasma gondii, ATP synthase forms distinct hexameric pyramids that are essential for maintaining their unique cristae morphology. Thus, ATP synthase physically shapes the membrane environment to optimize the proton motive force, ensuring that ATP generation remains both efficient and flexible [106, 107].

### Membrane potential independence

The architecture of cristae enables compartmentalization within the mitochondrion, allowing different regions to maintain distinct ΔΨm [30]. This structural separation supports localized bioenergetic activity that allows regions of the mitochondrion to function independently [108]. Such independence is vital for preserving energy production during cellular stress or localized damage, ensuring that mitochondrial output remains uninterrupted.

Maintaining separate ΔΨm across compartments allows mitochondria to dynamically adjust energy production in response to changing metabolic demands [109]. This adaptability is especially important in cells with fluctuating energy requirements, such as muscle or immune cells. It also protects against the spread of dysfunction; if one area experiences a drop in membrane potential, other compartments can continue operating effectively. This containment mechanism helps preserve mitochondrial function, sustain ATP production, and reduce the risk of cell death.

### Specific protein localization

The strategic positioning of OXPHOS proteins within the cristae is essential for efficient energy conversion [27, 62]. Proper spatial organization ensures optimal electron transport and proton gradient formation, both of which are crucial for maintaining mitochondrial function [110]. The MICOS complex, in coordination with ATP synthase and OPA1, helps shape and stabilize the IMM to promote effective bioenergetic processes.

This targeted protein localization highlights the sophisticated relationship between mitochondrial structure and function. By anchoring these complexes in specific regions, cristae enable efficient energy production while allowing mitochondria to adapt to changing metabolic demands to support cellular energy homeostasis.

### Expanded roles of cristae in metabolism and cellular functions

As the central sites for respiration and metabolism within mitochondria, cristae play a vital role in helping cells adapt to stressors like nutrient deprivation, oxidative stress, and DNA damage. Their intricate architecture not only supports ATP production but also enables mitochondria to manage metabolic byproducts, such as ROS and ammonia. Changes in cristae structure in conditions, such as heart failure and metabolic syndrome can impact mitochondrial efficiency and disrupt energy balance [111]. The strategic arrangement of protein complexes within cristae enhances the ability of mitochondria to respond to oxidative stress by facilitating detoxification and recycling of damaged components.

Cristae also act as sensors and responders to metabolic cues by continually reshaping in response to shifting energy demands. Under conditions of starvation, cristae can elongate and fuse to sustain ATP production efficiency and protect mitochondria from autophagic degradation. Conversely, in nutrient-rich environments associated with obesity or type 2 diabetes, cristae often appear swollen or fragmented, leading to cristae collapse that impairs respiratory supercomplex assembly and exacerbates ROS production [112–114]. This structural flexibility is essential for regulating metabolic and signaling pathways that are crucial for cell survival and proliferation [115]. Cristae also play a role in immune regulation through their dynamic and organized architecture [116]. For example, OPA1 is essential during thymocyte development. Its loss disrupts the metabolic programming of mature memory T cells. The example highlights the importance of mitochondrial dynamics in immune cell function [117].

Dynamic remodeling of cristae is especially important in cytotoxic T lymphocytes [CD8^+^ T cells], where changes in cristae organization influence mitochondrial energy output and signaling. These changes are essential for the rapid proliferation and activation of CD8^+^ T cells during immune responses [118]. By modulating mitochondrial bioenergetics, cristae directly shape how immune cells respond to pathogens and carry out their defensive roles.

Ongoing research continues to uncover how cristae remodeling influences both health and disease. These findings reflect the essential role of cristae in mitochondrial function and cellular adaptation.

### Cristae as structural barriers for mitochondrial compartmentalization

Cristae create highly specialized, compartmentalized environments that enhance energy conversion while regulating the spatial separation of metabolic processes. This organization helps prevent interference between reactions and supports the overall stability and efficacy of the mitochondria. In this section, the dual role of cristae as a functional platform and a physical barrier for compartmentalization within the mitochondrial matrix will be discussed.

### Formation and maintenance of cristae compartmentalization barrier

As discussed earlier, the IMM is stabilized by specialized lipid scaffolds enriched in conical phospholipids to support its curved geometry. These structural features are connected through the MICOS complex to produce CJs that define the compartmentalized structure of cristae. The resulting lipid–protein frameworks form functional barriers that organize mitochondrial space and help maintain bioenergetic efficiency.

These compartments are critical for minimizing interference from surrounding metabolic processes. By restricting the diffusion of ROS and other potentially harmful by-products, they protect mitochondrial and cellular integrity. Cardiolipin preferentially localizes to the inner leaflet of the cristae membrane, contributing to the formation of distinct zones that support efficient OXPHOS by spatially segregating major metabolic reactions [119]. The interactions between these lipid scaffolds and respiratory super-complexes not only preserve cristae structure but also enhance the functional performance of the mitochondrial respiratory chain [120–122].

### Compartmentalization to enhance metabolic efficacy

An important advantage of cristae compartmentalization is the precise localization of enzymes and protein complexes within distinct regions of the IMM. This spatial organization enhances the coordination of biochemical reactions that improves metabolic efficacy and boosts the integration of multiple energy-producing pathways.

The architecture of cristae is designed to concentrate enzymes and substrates within confined domains. This arrangement is responsible for streamlining interactions among metabolic processes, such as the citric acid cycle, OXPHOS, and ATP synthesis. The setup enables rapid transitions between metabolic steps and promotes the assembly of respiratory super-complexes, which improve electron transport efficiency and reduce the diffusion distance for metabolic intermediates [123]. This optimized configuration minimizes energy loss and enhances the overall performance of mitochondrial respiration by tightly channeling energy within the mitochondrial matrix [107].

Most strikingly, recent groundbreaking research has highlighted how cristae architecture can drive metabolic heterogeneity within a single cell. Ryu et al. demonstrated that cellular ATP demand segregates mitochondria into two metabolically distinct subpopulations based on the presence or absence of cristae. When cells are reliant on OXPHOS, the pyrroline-5-carboxylate synthase (P5CS) which is essential for the reductive biosynthesis of proline and ornithine, sequesters into a specific subset of mitochondria that lack cristae and ATP synthase. These cristae-deficient mitochondria are dedicated to reductive biosynthetic pathways, structurally separated from the cristae-rich mitochondria that perform oxidative phosphorylation. This segregation is regulated by mitochondrial fusion and fission cycles, preventing competition between oxidative and reductive processes. This finding provides the definitive proof that the presence of cristae serves as a structural switch, compartmentalizing mutually exclusive metabolic programs into distinct organelles [124].

By fine-tuning the spatial arrangement of enzymes and metabolic intermediates, cristae act as hubs that support fast, efficient energy production. Their architecture exemplifies the profound connection between form and function in biology, highlighting how structural organization enables mitochondria to power vital cellular activities with precision and speed.

### Protective effect of compartmentalization on stability

Beyond bioenergetic functions, the compartmentalized architecture of cristae serves as a fundamental defense mechanism essential for safeguarding mitochondrial integrity. A primary function of this spatial organization is the protection of the mitochondrial genome. The mtDNA is highly susceptible to oxidative lesions due to its proximity to the ETC. However, recent super-resolution microscopy reveals that cristae architecture enforces a physical segregation between the genetic material and the sites of ROS generation. While ROS-producing ETC complexes are densely packed within the cristae membrane, mtDNA nucleoids are predominantly anchored to the inner boundary membrane or at the base of CJs. This spatial separation creates a protective niche that shields the genome from the high flux of superoxide radicals generated at the cristae tips, thereby minimizing mutation rates and preserving transcriptional fidelity [125, 126].

Furthermore, CJs act as selective diffusion barriers that are critical for preventing accidental cell death. The intracristal space stores the bulk of cellular cytochrome c. Under homeostatic conditions, tight CJs maintained by the OPA1-MICOS axis physically sequester cytochrome c within the cristae, preventing its leakage into the intermembrane space where it could trigger apoptosis [32, 80]. During metabolic stress, this barrier function ensures that transient fluctuations in membrane permeability do not escalate into a catastrophic release of apoptogenic factors.

**Fig. 5:**
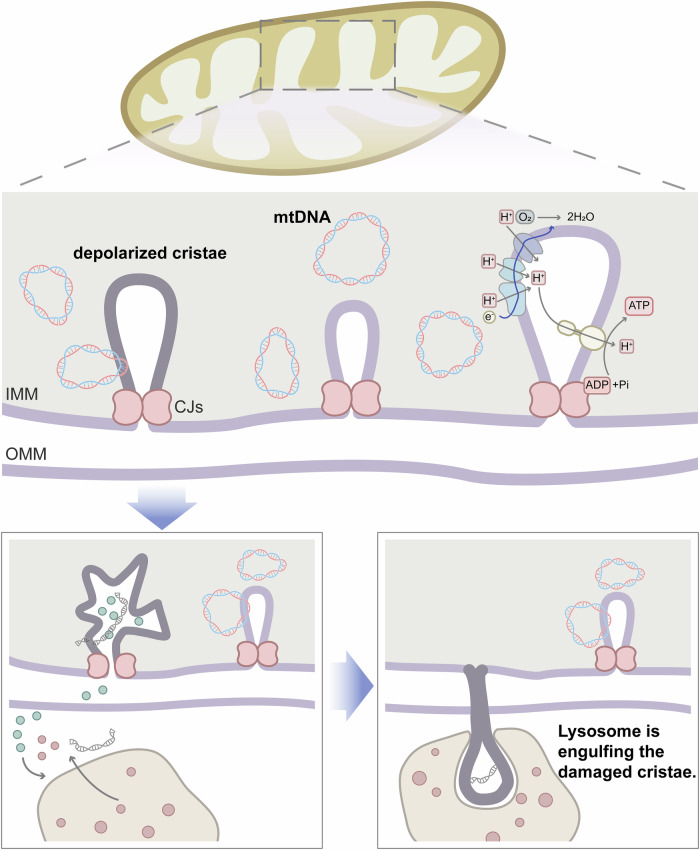
Cristae compartmentalization enables damage containment and localized quality control. The architecture of the IMM creates bioenergetically independent units, preventing the spread of dysfunction. This compartmentalization allows a specific crista to undergo depolarization (left, gray loop) while neighboring cristae remain fully polarized and functionally active (right), continuing to generate ATP via the electron transport chain. The structural isolation of damaged cristae facilitates their selective removal. Instead of triggering whole-organelle degradation, the impaired cristae and their contents are sequestered and engulfed by lysosomes, serving as a built-in quality control mechanism to preserve overall mitochondrial homeostasis.

Finally, this compartmentalization enables a sophisticated “damage containment” strategy. Individual cristae operate as functionally independent units; if a specific crista is irreversibly damaged by oxidative stress, it can be structurally isolated from the remaining healthy network. Recent evidence demonstrates that these damaged cristae—along with their oxidized proteins and lipids—can be budded off into vesicles derived from the IMM (VDIMs) for selective lysosomal degradation (Fig. 5)[127]. This mechanism allows mitochondria to amputate dysfunctional components without destroying the entire organelle, thus maintaining overall mitochondrial homeostasis and lifespan [128].

### Conclusions and future perspectives

The architecture and function of cristae reflect an evolutionary innovation deeply rooted in the ancestral origins of mitochondria as bacterial endosymbionts. Structurally, cristae bear a striking resemblance to mesosomes—invaginations of the bacterial plasma membrane that enhance enzymatic capacity for respiration [129–131]. Like cristae, mesosomes form distinctive intracellular compartments under stress, generating endogenous hydrogen peroxide [132–134]. These structural parallels, combined with the conservation of cardiolipin-rich lipid scaffolds and bacterial-like protein organizations (e.g., MICOS and OPA1), suggest that cristae represent a specialized adaptation of these prokaryotic precursors. By retaining these ancestral traits, cristae safeguard the mitochondrial homeostasis and sustain efficient ATP synthesis, mirroring how bacterial membranes protect cells under diverse environmental conditions.

However, identifying these evolutionary roots is only the beginning. To fully harness this knowledge for biomedical advancement, future research must transition from static structural descriptions to dynamic functional mechanistic studies. We propose four key frontiers for the next decade of cristae research:

(1) From static structure to 4D dynamics: emerging advances in imaging are revolutionizing our view of cristae. While electron microscopy provided static snapshots, the future lies in live-cell super-resolution nanoscopy (e.g., STED, SIM) and in situ Cryo-electron tomography. These technologies will allow researchers to visualize how this bacterial-like remodeling traits operate in real-time within eukaryotic cells—specifically, how cristae open and close within milliseconds to act as metabolic switches during rapid physiological changes.

(2) Deciphering tissue-specific heterogeneity: cristae regulation is not uniform; it is highly context-dependent. Understanding why cristae architecture differs between energy-storage tissues (adipose) and energy-demanding tissues (heart/brain) will be crucial for developing organ-specific metabolic therapies.

(3) Expanding the organelle crosstalk network: cristae do not function in isolation. A critical emerging area is the communication between cristae junctions and other organelles, particularly the endoplasmic reticulum at mitochondria-associated membranes. Investigating how stress signals from the endoplasmic reticulum are transmitted across the outer membrane to directly influence MICOS stability and cristae curvature will shed light on integrated cellular stress responses.

(4) Targeting cristae for precision medicine: finally, insights into cristae mechanisms must guide the development of new therapies. Moving beyond general antioxidants, the field should focus on structure-based drug design targeting specific cristae components. Developing molecules that allosterically stabilize the OPA1-cardiolipin interaction or mimic the membrane-shaping properties of MICOS offers a promising strategy to reverse mitochondrial dysfunction in neurodegenerative and metabolic diseases.

Together, these advances highlight the deep evolutionary link between prokaryotic systems and contemporary energy demands, promising a new era where cristae biology drives innovations in cellular bioengineering and precision medicine.
